# Extracts

**Published:** 1882-03

**Authors:** 


### Extracts.

A SO CALLED HERMAPHRODITE.—There is now in London a young Frenchman who presents peculiarities alike interesting physiologically and anatomically, and distressing socially. The child of healthy Parisian parents, there was nothing peculiar noticed about him as an infant; he was believed to be a female, and named, registered and dressed accordingly. At about the age of eight years some suspicion arose that the genital organs were peculiar for a girl, but no special notice was taken. As a child he played more freely with his brothers than his sisters, and liked bicycling and riding horseback and boyish sports. Later he went to a girl's boarding-school, where he staid till sixteen years of age. He is now twenty and dresses as a woman, and his appearance in that attire is not in anyway striking. The face is smooth and plump, with no signs of beard or whiskers, but a very few fine and short hairs are growing from the extremities of the upper lip as in a boy of fourteen. The growth of hair from the scalp is luxuriant and reaches below the waist. He asserts that no means of stimulating this growth has been adopted, and speaks of the trouble of constantly having to cut it. The voice is neither markedly masculine nor feminine. The larynx is small, the thyroid cartilage is not prominent, and has all the outward character of that of a woman. The mammæ are well, not largely developed, and glandular structure is distinctly to be felt; the nipple is fairly well formed and pinkish in color. There is a very scanty growth of dark hair on each axilla. The pubic hair is abundant, but does not extend up toward the umbilicus in the middle line. There is a distinct penis about two inches long, with a well-formed glans, and the meatus urinarius is represented by a dimple. The prepuce forms a kind of cowl over the glans, and is wanting below. The scrotum is completely divided along the middle line, and in each half is a well-developed testicle, with a normal epididymis and spermatic cord. On separating the two halves of the scrotum the opening of the urethra is exposed at the root of the penis, funnel-shaped, and easily admitting the little finger as far as the membranous urethra; a catheter passing readily along it into the bladder. On the inner side of each scrotal half, and close to the orifice of the urethra, is a muco-cutaneous fold very much like a nympha. The perineum is long, like that of a male. By rectal examination the outline of the prostate is readily defined. The thighs and legs are distinctly of the masculine type, as is the gait; the arms are those of an unathletic man, and the hands are large. He has good health, but requires a great deal of exercise to keep well. He asserts that he has no exclusive preference for either sex, and that he has been in love with both men and women. There is the usual evidence of functional activity of the testicle. The associates of this man speak of his general demeanor as masculine, though occasionally markedly feminine, and they often say of him that "she is a good boy." Of course there is no question as to the sex of this individual; he is a man with hypospadias. The special features of his case are the development of the breasts, the growth of hair from the head, the hairless face and small larynx. It is not strange that in infancy he should have been taken for a girl, for with the smaller size of all the external genitals, the resemblance to the female sex must have been very close. It is an interesting question how far the resemblance in character to woman—such as it is—is due simply to his education and constant association with women, or how far it is dependent upon some original peculiarity similar to that seen in the development of the mammæ and hair.—*Lancet.—St. Louis Med. and Surg. Journal.*

---

BURIED AT SEA.—On the steamer Parthia, which reached New York September, 14, Professor William Warren Greene, of Portland, Me., sailed from England. He had gone with other prominent physicians to attend the International Medical Congress at London, which was held in August, and at it he had on several occasions put forth opinions on surgery which called for the learned consideration of his colleagues. His remarks on the causes of failure in obtaining union in operation wounds and on the method best calculated to secure it had been preserved. Long a sufferer from Bright's disease, Professor Greene was unable to continue his stay in England, and as his symptoms took the appearance of uræmia, which he felt would be fatal, he sailed for home. Drs. Sayre and Little were fellow passengers, and Dr. Gamble, the medical authority aboard the Parthia, lent his aid to their counsel and treatment. Professor Greene, however, sank rapidly, and on the morning of the 10th inst., while the Parthia was in latitude 43 deg. 40 min. north, longitude 49 deg. 28 min. west, he expired. As the vessel was yet remote from land it became imperative to give the body a sea burial. All the passengers—and the Parthia carried more on this trip than ever before—joined in the expression of regret at the Professor's demise, and seemed deeply impressed with the services. At four o'clock in the afternoon the crew, in uniform, were mustered on the upper deck, on which the passengers had already gathered. There were several clergymen aboard ship, but according to the rules of the Cunard Company and the practice of the service, the captain of the vessel officiated. The ensign floated at half mast, and the engines of the Parthia were slowed, so that she lay almost still upon the waters underneath a clear sky and in weather which was pleasant beyond precedent this season. Presently the bells fore and aft began to toll, and then, borne by six seamen and covered by the American flag, the coffin containing the remains of the deceased was borne to the gangway. It was the usual kind of casket used at sea, with iron weights and perforated sides to insure its sinking to the bottom. Then all heads were uncovered and Captain McKoy took his place at the gangway. A beautiful hymn adapted to the solemn nature of the occasion was sung by all on board, and then the impressive burial service of the Church of England was read, This done the bells tolled again, and the coffin was committed to the sea.—*Gaillard's Journal.*

---

Extravasation of Urine.—A few words now with regard to the manner in which this accident occurs. The urethra is studded with little follicles or racemose glands, some of which are at the bottom of a little canal, or canaliculus, and from these the lubricating fluid of the urethra is in great measure derived. There is notably one found in almost all urethræ, on the superior surface of the canal, about half an inch from the meatus externus, the lacuna magna, which is often large enough to admit the end of a small sized probe. These little secreting glands at the bottom of depressions, which are more numerous in some urethræ than in others, sometimes become inflamed independently of inflammation of the surrounding tissue. That is to say, there sometimes occurs trouble localized in a single one of these depressions, and when this is the case, it is usually the result of a stricture of large calibre; a stricture which is just sufficient to form a slight obstruction, behind which pus or other material may lodge and keep the parts in an abnormal condition, ready to take on inflammation. Inflammation having been thus excited in one of these follicles, suppuration takes place, pus burrows along, sometimes a distance of half an inch or an inch; sometimes it makes its escape externally in the shortest direction. In any case we have a passage formed through the urethra into the tissues, and into this urine readily finds its way, sometimes, however, in so small quantity that it simply gets up a little accumulation of plastic material, which finally forms a small nodule and stops the further progress of the trouble. Again, the opening may be larger, and urine may find its way into it in greater quantity, and cause active inflammation in the tissues, forming an abscess, through which extensive infiltration may take place.

I insist upon it that no difference exists in the nature of the two cases, and whenever a swelling of the perineum occurs independent of an immediate injury, or a furuncular trouble, we may be sure that in ninety-nine cases out of a hundred it is the result of follicular penetration of the urethral canal, from the causes previously stated, and that it will go on to the formation of an abscess of greater or less size; and we cannot tell in any case, whether what appears to be a small affair is going to remain so, or whether it is going on to the destruction of a large amount of tissue. Some of the most innocent appearing cases have gone on rapidly, within a few hours, to result in infiltration of urine and speedy death to the patient.—*Fessenden N. Otis, M.D., in Med. and Surg. Reporter.*

---

Alcohol in Therapeutics.—In a course of addresses on "Abstinence," delivered before the Hunterian Society, of London, in 1878, Dr. Benjamin W. Richardson uses these words in referring to the difficulties that surround the use of alcohol in general medical practice: "But prescribe it as a medicine; do not permit its use as a beverage. Prescribe it as alcohol from the dispensary. Learn the exact quantity that is required to produce the desired effect, and then you will discover, and in no other way, whether the good attributed to the grog is due to the alcohol it contains, or to some other agency." This is the scientific, and, as I think, the common sense mode of procedure.

Dr. Richardson sounded the key note when he advocated the use of alcohol in medical practice in lieu of beer, wine, whiskey, and other liquors containing alcohol. Within the past two years the medical officers of the Inebriates' Home, of Fort Hamilton, N. Y., have recognized the value of Dr. Richardson's suggestions, and adopted them in the treatment of the various forms of alcoholism, when the use of alcohol was indicated, and have found the following benefits to proceed from their adoption:

1st. The satisfaction of having a solution containing a definite per centage of alcohol, thus providing an exact system of dosage.

2d. A very marked saving, and, therefore, a method of value from an economical point alone, especially in those institutions where the liquor bill is a large item of expense.

3d. The moral effect on the patient, in compelling him to at once break off his accustomed stimulant and providing a "medicine" as an efficient substitute.

4th. The advantages which the alcohol has over brandy, whisky, etc., is that, owing to accuracy and concentration of the dosage, the patient convalesces in one-half the time and with less suffering.—*Dr. L. D. Mayson*, *in Med. Record.*

---

A Successful Operation of Gastrotmomy for Intussusception.—On the 3d day of October, 1881, Oscar Holly —a strong, robust, muscular man—was suddenly seized with a severe abdominal pain, of a griping or spasmodic character, referred to the neighborhood of the umbilicus. My father, Dr. J. M. Estill, was immediately summoned, who exhausted all the known remedies for mechanical obstruction of the bowels, viz.: catharsis, copious enemata, opium, etc., without result. He continued to treat the patient upon general principles, until Saturday morning —five days after his attack began—when, according to previous arrangements, he started for the meeting of the Medical Association of Virginia at Old Point Comfort, and I, who had been absent in the meantime, saw him, at 2 o'clock on Saturday morning, for the first time. I found him with coldness of the skin, prostration, distressed countenance, persistent constipation, constant vomiting entirely of a stercoraceous character, abdomen tender and very tympanitic, with a slight tumor, dull upon percussion, immediately to the left of the umbilicus. From these symptoms, and the history of the case, I diagnosed intussusception or invagination, and decided that an operation was the only remedy by which to give my patient a chance for life.

Accordingly, on Monday morning, the 10th day of October, and the 8th day of Holly's illness, I, with the kindly assistance of my friends, Drs. A. S. Huffard and Thomas Witten, performed the operation of gastrotomy, opening the abdomen between the umbilicus and pubes, along the linea alba, five inches, through the peritoneum; and passing my fingers to the supposed point of obstruction, I found the diagnosis to be correct, the invagination occurring in the small intestines (jejunal) immediately to the left of the umbilicus. The gut *below* the obstruction was very much engorged, and presented every appearance of acute enteritis. The adhesions were pretty firm, but with moderate force they were separated. The wound was closed with sutures, the peritoneum being included; but, on the fourth day, as peritonitis had supervened, the sutures were removed and plaster substituted therefor. The peritonitis very readily yielded to treatment; the temperature did not exceed 100° F., and the pulse did not reach 100. I should have mentioned that the antiseptic method (Lister's) was used throughout.

On this, the 22d day after the operation, the patient is able to walk, and in a few days will be able to engage in his usual avocation.—*H. B. Estill, in Va. Med. Monthly.*

Pulmonary Hemorrhage.—There are two or three things employed for this purpose that are mainly relied on just now for the suppression of the bleeding. First, the fluid extract of ergot; and I believe it is as efficacious as any medicine we can give internally. But there is something still more efficacious. By temporarily stopping, somewhere in the system, a certain quantity of blood, and holding it there for a few minutes, the effect of which is to relieve the lungs. When the hemorrhage is moderate, dry cups serve this purpose; a dozen of them may be applied, they hold the blood in the capillaries and keep it out of the general circulation so long as they are applied. I was attending a case of effusion at one of the hotels here, and a case of as obstinate a hemorrhage as I have ever seen. It was in a young man, a merchant's clerk, and he was running down very rapidly. The flesh was melting away from him, he would have hemorrhages every day, perhaps two, sometimes three. Though each one could be stopped, the occurrence was so frequent, that he was losing life. Dr. Detmold was called in consultation, and seeing the danger of the case said, "why not resort to the same plan that is used in the army, for stopping hemorrhages in persons who have been shot through the lungs?" I said "yes, certainly." "Well," said he, "let us tie up one of his arms as if for bleeding." A bandage was put about one of the arms, and the blood accordingly accumulated in the veins so that the hands and arms were swollen; when the arm filled with blood the hemorrhage stopped. We held the blood there in the veins for perhaps four or five minutes and then tied the other arm and allowed the blood from this to flow out into the general circulation, and in perhaps ten minutes we left him to himself and the bleeding did not occur that day again.

The next day there was but little hemorrhage. Whenever the hemorrhage was dangerous, he was instructed to have the arm tied up in that way and he got well enough to go back to his work. While he made but slow progress, he was able to work two or three years after that, then he went to Europe and I don't know but that he died there. I have resorted to that expedient in a great many cases since and have never found it fail as a temporary relief. Do not allow the blood to remain in the vessels long enough to coagulate there. Not knowing exactly in what time it would coagulate, I prefer to liberate the blood in one arm after the ligature has been applied for five minutes, and if necessary tie up the other arm or tie a leg; confine a portion of blood out of the circulation for a little while and the hemorrhage will almost invariably stop.—*Alonzo Clark, M.D., in Med. Gazette.*

---

WHAT IS RESORCINE ?—Resorcine, says the Journal of Chemistry, belongs to a class of bodies called phenols, to which carbolic acid also belongs. In chemical composition they resemble alcohols. Pyrogallic acid is a triatomic phenol.

Resorcine was originally obtained by fusing certain resins, as gum ammoniacum or galbanum, with caustic potash, extracting it from the fused mass by acidifying with sulphuric acid and shaking with ether, and then purifying it by distillation.

Resorcine crystalizes in colorless plates or columns, and dissolves readily in water, alcohol and ether. It melts at 104° Cent. (219° F.) and boils at 271° Cent. (521 F.,) and can be obtained perfectly pure by distillation. It is claimed that it can be made in Switzerland for four dollars per kilogram (about one dollar and eighty cents a pound,) but we find it quoted in German price-lists at from seven dollars and fifty cents to ten dollars per kilogram, or about double the price of salicylic acid. To some extent the price will depend upon the demand.

Resorcine is not poisonous in moderate doses. From twenty-five to thirty grains is required to produce any marked effects. It has been found to reduce the temperature in febrile complaints, but its effect is of short duration, and unpleasant after-effects have been noticed. One of its isomers—hydrochinone—is preferred for use in fevers, the dose being smaller. The third isomer—pyrocatechine—has powerful toxic properties.

Andeer has recently investigated the properties of resorcine, and finds that it possesses the power of stopping decay. A one per cent. solution of chemically-pure resorcine will stop the development of fungi and mold. In every degree of concentration it coagulates albumen and precipitates it from solution, on which account it may be used as a caustic to remove unhealthy tissue. In crystals it cauterizes as powerfully as lunar caustic. In minute quantities resorcine will preserve ink and colors, which would otherwise mold quickly, and does not injure the color. To stop fermentation completely requires a rather strong solution of one and a half to two per cent.

Dr. Koller prophesies a great future for resorcine, which, he says, will be the disinfectant and antiseptic of the physician, the druggist and the chemist. It must not be forgotten, however, that resorcine is too powerful a reagent to be taken internally in unlimited amount.—*Phil. Med. Times.*

---

External Hemorrhoids.—As I separate the buttocks of this man, you will be able to see upon the right side of the anus an external pile, or hemorrhoid. The condition is a very simple one, it is nothing more than a clot of blood, which has been thrown out under the skin among the subcutaneous cellular tissues from the rupture of a hemorrhoid vessel. It is due, in the vast majority of cases, to straining efforts while at stool, during a condition of constipation. The passage of hardened fecal matter is always irritating to these little tumors, causing them to be more or less congested and frequently acutely inflamed. They are always the source of a great deal of pain and annoyance, and frequently incapacitate a person for business, or even to walk about or sit quietly in a chair with any degree of comfort.

This little tumor has only existed since yesterday, and I can scarcely say whether it is yet ripe or not. If an external hemorrhoid is opened before it is ripe, troublesome hemorrhage is very apt to ensue. And the means necessary to control this hemorrhage would only increase the severity of the already existing condition of affairs.

What I mean by an external hemorrhoid being ripe, is that the clot should be well formed. This point can always be ascertained by carefully examining the density of the tumor. If it is firm, hard and full like a shot or bullet, when between the fingers, it is perfectly safe to remove it; but if it is soft and easily compressible it had better be allowed to remain and ripen.

When these tumors are small and recent, the local application of the compound ointment of galls answer very nicely, but when they are older some operative procedure must be instituted. The old way of treating an external pile, by cutting it off with a pair of scissors, and then allowing the parts to heal by the granulating process, is a barbarous mode of procedure; and the new way of treating them, by the hypodermic injection of strong carbolic acid, can scarcely be any better. By far the more satisfactory plan is to lay the tumor open, turn out the clot and make local applictions of lead-water and laudanum or Goulard's extract. This is what will be done in the present case.—*Prof. S. W. Gross, Clinical Lecturer, in Med. and Surg. Reporter.*

---

THE DEVELOPMENT OF A SINGLE BREAST IN GIRLS.—M. Despres took occasion of the presence of a girl at his clinic to draw the attention of his class to a circumstance that causes alarm to mothers, and is sometimes judged wrongly of even by physicians. This was an example of the development of only one breast at the age of puberty, when the belief is often entertained that this arises from the presence of a tumor. This girl was thirteen years of age, and was brought to the hospital under the idea that she had a tumor of the right breast, the left one not yet having undergone any change. Her attendant had prescribed iodide of potassium. M. Despres at once assured the mother that it was only the natural development of the organ, and would be soon followed by the appearance of the menses and the development of the other breast. He observed to his class that while it is natural for mothers to be deceived in these cases, it should be impossible for the surgeon to be so. In fact there exists under the breast a regular prominence in the form of a movable disk on the chest, without the slightest adherence to the skin, and accompanied by no pain whatever. The nipple is exactly in the center of the tumefaction, and although the developing gland is resistant, it is never irregular and never presents lumps. A tumor of new formation, such as sarcoma, is always harder, and is never found exactly in the center of the mammary region.—*Gaz. des Hop.—Louisville Med. News.*

---

Carcinoma of the Breast.—From all the data contained in the paper, I believe that I am warranted in framing the following practical conclusions:

1. That surgical intervention in carcinoma of the breast tends to retard the progress of the disease by preventing local dissemination, implication of the associated lympathic glands, and the development of visceral tumors.

2; That local reproductions do not militate against a permanent recovery, provided they are freely excised as soon as they appear; and that lympathic involvement does not forbid operation, since infected glands were removed in more than one-third of the examples of final cure.

6. That the subjects are almost without exception safe from local and general reproduction if three years have elapsed since the last operation.

4. That the risk from operations is outweighed by the benefits which accrue from them, since they not only add twelve months to life, but also cure more than one half as many patients as they destroy.

5. That all carcinomata of the breast, if there are no evidences of metastatic tumors, and if thorough removal is practicable, should be dealt with as early as possible by amputating the entire mamma and its integuments, dissecting off the subjacent fascia, and opening the axilla, with a view to its exploration and the removal of any glands which were not palpable prior to interference.—*Samuel W. Gross, M.D., in Medical News.*

---

Chloroform to Sleeping Persons.—The following occurs in a review, in the *Journal of Medical Sciences*, of Artificial Anæsthesia and Anæsthetics, by Henry M. Lyman,

M.D. Our own observation and experience lead toward an endorsement of the paragraph:

In regard to the possibility of the administration of chloroform to sleeping persons without wakening them, the author quotes from Dolbeau and from Drs. Cluness and Quimby, of this country, giving in all fifteen cases in which the attempt was successful and thirty-three in which it was unsuccessful. In one of these quotations we find the truth stated, that chloroform can be thus succesfully administered, but only by one skilled in using it. The author himself gives no summing up, strikes no balance between the successful and unsuccessful cases, and expresses no opinion on the subject which would aid an advocate in prosecution or defence.

---

NOTHING is worse than a vacillating physician, whom each notion, each wish of the patient, each suggestion of nurse or family, affects. Blown hither and thither by every breath, incapable of taking a broad view of the case, his treatment soon becomes as irresolute as himself, and directions and bottles accumulate with bewildering rapidity. The fewer drugs that are used the better; the greater decision with which drugs are used the better.—*DaCosta—Louisville Med. News.*

---

PRURITUS ANI.—Dr. Packard has found the following ointment succeed in relieving obstinate cases of pruritus ani in which the whole array of ordinary remedies had failed:

℞. Camphoræ,

Chloral hydrat, - - - aa ʒss.

Ungt. petroleii, - - - - ʒvij. M.

S. Ointment; apply frequently.

*Practitioner.*

---

STERILITY IN BOVINE TWINS.—It has been observed that when one of the twins is male and the other female, the latter is always sterile, there being a malformation of the generative organs. The question has been asked whether the same condition of affairs has been noted in the human race under similar conditions.—*Journ. de Med. et de Chirurg. Prat.*

Badgering Scientific Witnesses.—The *Medical Gazette* (January 7) calls attention to the scandalous practice which some attorneys have of brow-beating witnesses summoned as experts by the opposing party to the suit. Too often the impression which would be left on a person ignorant of the modus operandi of modern criminal trials, on attending a court of justice during such a trial, would be that the object of cross examination is rather to amuse the audience and to exhibit the superior badgering qualities of the interrogator than to further the ends of justice. One can scarcely imagine a more harrassing position for a self-respecting man with a fine sense of honor than to be compelled by the law to submit to this degrading style of examination, which often amounts to positive insult. The tendency of this habit of brow-beating is to render witnesses, men of scientific attainments, for example, whose testimony may be invaluable in establishing truth, sly of coming forward to illumine a difficult case by the light of their experience. Shall the law itself furnish a cloak under cover of which a man may with impunity reflect upon the veracity and private character of his fellow; emboldened from immunity, from punishment, to probe the irrelevant secrets of an individual's private life? Forbid it justice! Public opinion forbid it!—*Michigan Med. News.*

---

The Danger of Administering Anæsthetics Without Witnesses.—This subject has been written upon time and again, and physicians have been repeatedly warned not to give an anæsthetic to any one, but in an especial manner to avoid using them with females, unless in the presence of witnesses. Yet the advice is unheeded, and every now and then we hear of some unfortunate results from this negligence. This time it comes from the West. An old and prominent physician was visited by a beautiful young lady, for treatment for some sexual disorder. The lady belonged to the blue blood of the city, while the physician had been her grandparents' attendant, and had for years been the family physician. On one of her visits he informed her that an operation was necessary, and induced her to take some anæsthetic. Subsequently, not improving, she consulted another physician, who told her she was pregnant. In due time a child was born.

The lady made no effort at concealment, but in a short time brought an action for damages against her old doctor. He publicly denied the charge, but refused to go into court and do so under oath. After a few minutes' deliberation only, the jury brought in a verdict against the doctor, assessing the damages at fifty thousand dollars. The verdict was received with such open manifestations of approval from the judge, as well as the audience, the former publicly shaking hands and congratulating the young lady in court, as to leave no doubt of the sympathy of the public. The case is a sad one; whether guilty or not, this old and established physician has been ruined, socially and professionally, forever.

It should be made a rule, never to be broken, by every physician, when he commences the practice of medicine, to refuse absolutely to administer an anæsthetic to any woman alone, no matter what the collateral circumstances may happen to be.

Women are peculiar and unreliable, and there is no accounting for the queer notions they may sometimes get into their heads; so that no physician is surely safe from trouble who has passed a period alone with a woman who has been made unconscious by an agent administered to her by him.—*Med. and Surg. Reporter.*

---

Treatment of Cystitis —Dr. A. J. C. Skene, of Brooklyn, gives the following, which he regards as almost specific in its influence, especially in the earlier stages, affording rapid and lasting relief: R. Acidi benzoici, Sodii biboratis, aa grs. x.; Inf. Buchu, ℥ij. M. Sig. This quantity to be taken three or four times a day. The diet should also be carefully regulated, and the skin and bowels kept in active condition.—*Cincinnati Lancet and Clinic.*

---

New Theories.—In the *Chicago Medical Journal and Examiner*, Dr. Valin presents "a new theory of generation." How easy is it to cudgel the imagination (rather than the brain) into generating a new theory.

